# Monitoring Zoo Elephant Rumble Activity Using Combined Seismic and Acoustic Data

**DOI:** 10.1002/ece3.73220

**Published:** 2026-03-08

**Authors:** Fabian Limberger, Georg Rümpker, Ronja Wesemann, Abolfazl Komeazi, Tanja Spengler, Martin Becker

**Affiliations:** ^1^ Institute of Geosciences Goethe University Frankfurt Frankfurt am Main Germany; ^2^ Frankfurt Institute for Advanced Studies Frankfurt am Main Germany; ^3^ Opel‐Zoo, Education and Research Kronberg im Taunus Germany

**Keywords:** bioacoustics, convolutional neural networks, elephant communication, infrasound, non‐invasive sensing, seismic signals, wildlife monitoring, zoo animal behavior

## Abstract

The communication of African and Asian elephants based on seismic and acoustic waves has been studied for decades. However, research within anthropogenic zoo environments, particularly with respect to seismic signals, remains limited compared to studies in natural habitats. This study analyzes low‐frequency elephant rumbles recorded at the Opel‐Zoo near Frankfurt am Main, Germany, by comparing characteristics from datasets obtained using non‐invasive, co‐located seismic and infrasound sensors. Analysis of recordings from August 2024 revealed over 1350 rumbles, indicating significant temporal variability. These rumbles are characterized by signal durations of 1–8 s and fundamental frequencies between 10 and 25 Hz, with harmonics above. Due to high seismic background noise during zoo opening hours, infrasound detections are more abundant during the day, while seismic and infrasound detection rates are comparable at night. The systematic nocturnal housing schedule of the elephants leads to an increase in rumbling activity approximately every second night, with one pair showing substantially higher vocal communication than the other. Many rumbles occur in rapid sequences within minutes, suggesting elephant interaction or external triggers. Most rumbles are accompanied by motion‐induced signals associated with locomotion or trampling, phenomena not detectable with infrasound sensors measuring acoustic waves only. This highlights the value of combined seismic and infrasound data. To enable a robust automated classification of rumbles and noise for continuous monitoring, we train CNNs using spectrogram images of the hand‐picked seismic and infrasound rumbles as inputs. The models achieve up to 98% classification accuracy, while cross‐domain applications demonstrate better generalization and robustness of the CNN trained with seismic data. The seismo‐acoustic monitoring approach and resulting findings have the potential to enhance our understanding of zoo elephant behavior, social interactions, and welfare.

## Graphical abstract

At the Opel‐Zoo in Germany, we studied elephant rumble activity by recording low‐frequency signals with seismic and infrasound sensors. We detected over 1350 rumbles within 4 weeks that show temporal patterns, demonstrating the benefits of combining both sensing methods. This study provides insights into a promising tool for monitoring elephant activity, behavior, and potential communication in zoo environments.

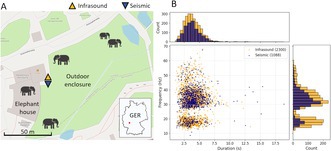

## 1 Introduction

For decades, scientists have used acoustic and seismic waves emitted by elephants to study their behavior and communication, as well as to monitor their movements. Non‐invasive measurement techniques have significantly advanced research efforts in elephant conservation and welfare, while also addressing challenges in human‐elephant conflicts to improve the protection and coexistence of both species.

Numerous studies have shown that elephants utilize both sound and seismic waves to communicate within their groups, particularly through rumble‐like signals that can be detected by seismic and infrasound sensors (O'Connell et al. 1997; Smith 2001; Günther et al. 2004; Poole 2011). Evidence from active playback experiments further demonstrate their ability to sense acoustic waves (Langbauer et al. 1991; Stoeger and Baotic 2017), but also purely elastic (seismic) waves (O'Connell‐Rodwell et al. 2006; Mortimer et al. 2021). Furthermore, Wrege et al. (2010, 2024) used non‐invasive methods such as seismic or sound sensors to monitor the impact of human activities like logging, gunfire, or oil exploration on elephant activity. In parallel Dissanayake et al. (2024) introduced an acoustic real‐time monitoring system and geo‐mapping tool. Garstang et al. (1995, 2014) showed that weather events and atmospheric controls could be sensed by elephants. In India, elephant‐human conflicts are an ongoing problem with danger for both species, e.g., due to frequent elephant and train collisions (Roy and Sukumar 2017). Hence, local and low‐cost monitoring systems to detect and track elephants based on the footstep signals became crucial and their development is ongoing (e.g., Anni and Sangaiah 2015; Chandan Chakraborty et al. 2024; Rithwik Vallabhanm et al. 2024).

In contrast to conventional bioacoustics, seismic waves and infrasound generated by elephants can operate at relatively low and inaudible frequencies, extending down to a fundamental frequency of 10 Hz. Such signals can be analyzed for various purposes: localizing elephants (e.g., Anni and Sangaiah 2015; Reinwald et al. 2021), assessing signal characteristics based on elephant traits like body size, gender, and vocalization styles (Soltis 2010; Poole 2011; Hedwig and Kohlberg 2024), or classifying signal types and patterns (Mortimer et al. 2018), even from diverse animals in natural habitats (Szenicer et al. 2021; Steinmann et al. 2025). Some of these studies used artificial neural networks for the detection (e.g., Costa et al. 2023) and classification of seismic signals, similar to approaches applied in bioacoustics, to estimate animal population based on detected vocalization numbers (Schneider et al. 2024). Moreover, recent studies suggest that elephants communicate through individual signature calls within their vocalizations, and that hierarchical structures influence group communication (Pardo et al. 2023; O'Connell‐Rodwell et al. 2024), further demonstrating that elephants are highly social animals.

In general, elephant communication is multifaceted, encompassing high‐frequency and low‐frequency sound waves, visual cues, body motions, chemical signals, and seismic ground motions. While sound wave‐based communication has been extensively studied, as presented by Stoeger (2021) or Narins et al. (2016), the characteristics, purpose, and propagation mechanisms of seismic waves generated by elephants remain poorly understood. Specifically, a systematic comparison between infrasound and seismic wave components of the signals is still lacking. Unlike sound waves, seismic waves are generally not limited to vocalizations; they can also arise from body motions such as locomotion and trampling, offering additional information absent in sound‐only recordings (Szenicer et al. 2021). Furthermore, seismic waves usually propagate more effectively through solid media, like rocks and soil, than sound waves in the air, having the potential for farther propagation, while the attenuation depends on the characteristics of the soil. Nevertheless, low‐frequency waves are generally less affected by small heterogeneities and attenuation in the subsurface. This property likely makes seismic waves a robust communication medium for elephants (Narins et al. 2016), though the extent to which elephants consciously exploit this advantage remains an open question. Reinwald et al. (2021) localized elephant signal sources and compared accuracy from higher‐frequency acoustic measurements and seismic measurements, suggesting that seismic signals are more suitable and accurate for localizations. However, the analysis of temporal patterns of rumble activity is missing.

Most existing studies on elephant seismic signals have been conducted in open natural habitats of wild African or Asian elephants. Nevertheless, controlled settings such as zoos offer a special research environment, as they provide stable conditions where observations can be more easily collected and constrained (Fernandez and Timberlake 2008). Elephants kept in zoos are exposed to anthropogenic factors such as visitors, facilities, routines, and surrounding traffic, which reduces the general comparability between elephant activity and behavior in zoos and natural habitats. The fact that many zoo elephants were born in zoos and have never been in their natural environment underscores this necessary distinction. So far, just a few recent seismic studies were performed in controlled zoo settings, e.g., by Vidunath et al. (2024) in Sri Lanka and Zetterqvist et al. (2023) in Sweden, focusing on method development for detecting ground motions by rumbles or footfalls and wave arrival directions. Nevertheless, these studies have not presented any temporal activity patterns nor have they provided a comparison between infrasound and seismic data. This lack of studies is surprising, since combining seismic and sound observations of both vocalizations and locomotion might help to better monitor and understand their interactions, welfare and potential responses on external seismic noise sources in zoos in general.

To address these research gaps in comparing low‐frequency sound and seismic signals produced by elephants in a zoo environment, we analyzed more than 1350 rumbles recorded within a period of 30 days using single co‐located infrasound and seismic sensors at the Opel‐Zoo near Frankfurt, Germany. The aim of the study is to better detect and understand low‐frequency signals from elephants in a zoo environment affected by anthropogenic influences and to test a robust rumble detection tool. Furthermore, by leveraging this dataset, we identify temporal patterns in rumble activity and demonstrate the benefits of integrating infrasound and seismic data in noisy environments, such as zoos. This study establishes a promising approach and dataset, serving as the foundation for future investigations into non‐invasive wave‐based monitoring of elephants in zoo environments.

## 2 Material and Methods

### 2.1 Data Acquisition and Processing

**FIGURE 1:**
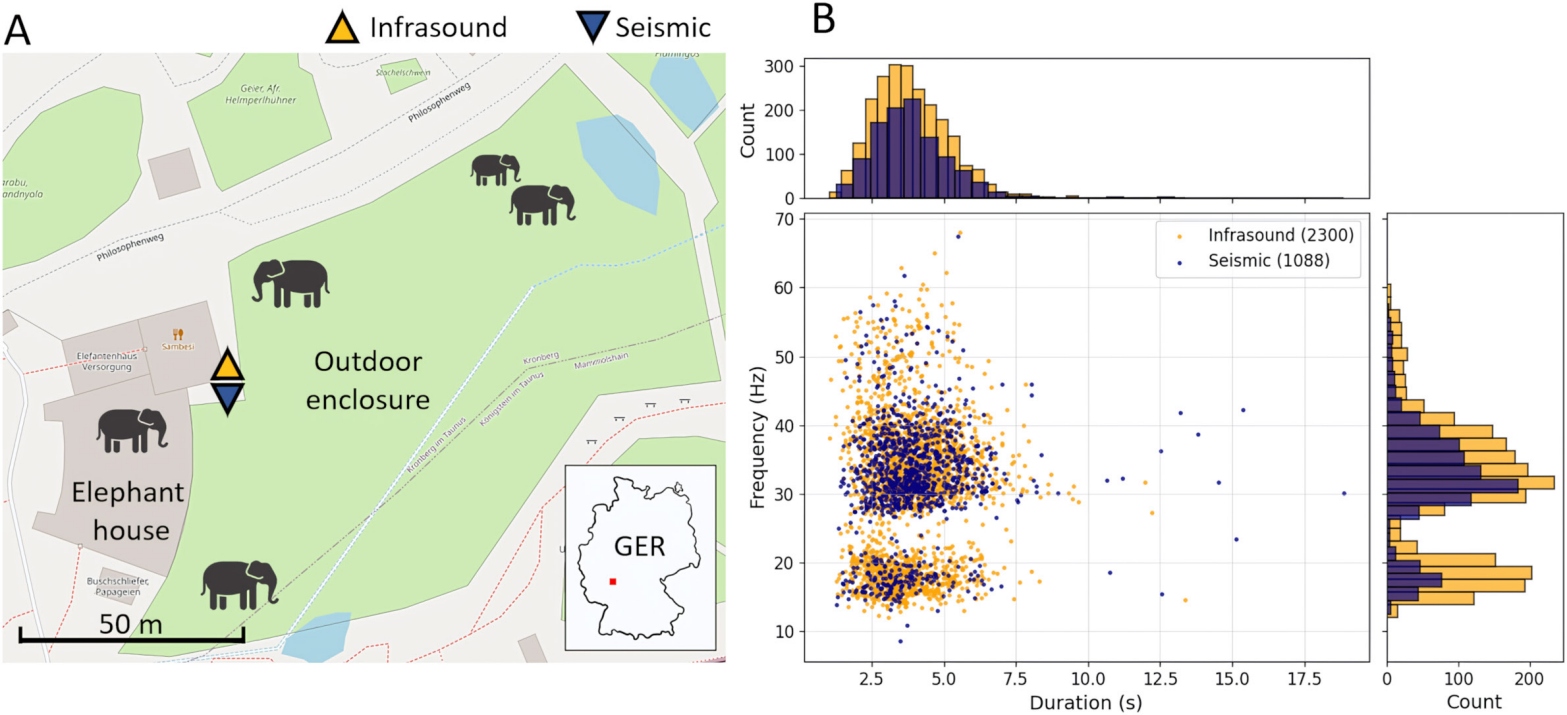
(A) Location of Seismic Sensor (Trillium Compact Post‐Hole 20s) and Infrasound Sensor (IST2028) nearby the elephants' enclosure at the Opel‐Zoo in Kronberg im Taunus, Germany (GER). (B) Distribution of frequency and duration of events detected on seismic and infrasound data (Image in A: OpenStreetMap).

The measurements for this study were conducted at the Opel‐Zoo in Kronberg im Taunus, near Frankfurt am Main (Germany), where five African elephants ( *Loxodonta africana* ) are housed. The group consists of one adult bull named Tamo, two adult cows Kariba and Lilak, and cow Cristina with her male calf Neco (see photos and information in the supplements). Tamo is mostly separated from the others, while Lilak and Kariba form one subgroup, and Cristina and Neco form another. The enclosure spans an area of approx. 10,000 m^2^ including a smaller indoor enclosure (Figure 1A).

For data acquisition, a three‐component seismic sensor (Trillium Compact 20s) was placed near the enclosure, complemented by an infrasound sensor (IST2018) measuring pressure changes, mounted on a nearby pylon attached to a building (Figure 1A). Both sensors operated at a sampling rate of 200 Hz, enabling the detection of infrasonic and seismic signals relevant to elephant rumbles and locomotion. The seismic sensor was deployed at the surface and covered with a plastic bucket fixed to the ground to shield it from direct acoustic‐wave coupling with the instrument. The frequency response of the IST2018 sensor spans corner frequencies from 0.05 Hz to 50 Hz, well suited for detecting low‐frequency seismic signals. The seismic sensor covers a frequency range from 0.05 Hz up to the Nyquist frequency of 100 Hz.

Continuous data collection spans 30 days, from August 2 to August 31, 2024. The collected raw seismic data (vertical component) and infrasound pressure data are bandpass filtered to 1–80 Hz and converted into spectrograms, which are scanned visually in 200‐s intervals to detect rumbles. To improve the visual detectability by increasing the signal to noise ratio, the spectrograms of the three seismic components (N, E, and Z) are averaged.

**FIGURE 2:**
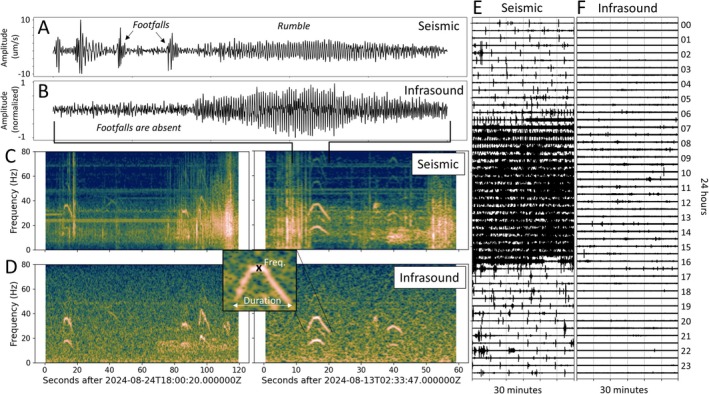
Comparison of seismic and infrasound recordings in both time and frequency domains. The seismogram (A) contains locomotion signals from footfalls and rumbles recorded as ground motions, while the infrasound recording (B) contains the signal of the rumbles only. Spectrograms of seismic (C) and infrasound (D) data from two exemplary time windows. The inset in D illustrates the parameters used to analyze an individual event, including the frequency at the apex, the signal duration, and timestamp. Footfalls are absent in infrasound data (B); however, rumbles are clearly detectable due to lower background noise conditions (D). Exemplary day plot of seismic (E) and infrasound data (F) shows increased noise during zoo opening hours. In particular, the noise increase during daytime is significantly higher regarding seismic recordings.

Then, rumbles are identified manually on both the seismic and infrasound spectrograms individually. For each rumble, a set of features is extracted, including the timestamp, frequency at the apex of the rumble shape, duration, and the label indicating whether it is detected from infrasound or seismic data (see Figure 2). Here, an “event” is defined as a specific combination of frequency and duration; hence, a single rumble can comprise several single event detections (e.g., fundamental frequency and first harmonic). For example, a rumble that is detectable by its fundamental vibration and two further harmonics consists of three events.

**FIGURE 3:**
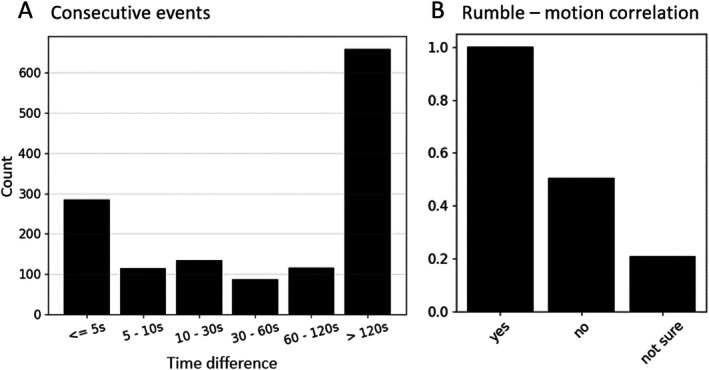
(A) Distribution of rumble time differences across six categories (time intervals). (B) Correlation between rumble counts and seismic signals associated with activities such as locomotion and trampling. A rumble is classified as ‘yes’ if a motion‐typical signal is detected within ±50 s of the rumble, ‘no’ if the rumble is isolated, and ‘not sure’ if the identification of a motion‐typical signal within the rumble time window is unreliable.

To approach potential communication patterns among the five elephants housed in the zoo, we furthermore analyze the occurrence and structure of consecutive rumbles. This is done by counting the number of rumbles with a time difference of < 5 s, 5–10 s, 10–30 s, 30–60 s, 60–120 s, and > 120 s (Figure 3A). These time periods are chosen to differentiate between immediate sequences (e.g., in case of direct communication) and sequences with longer durations to investigate the most probable time passing between rumbles.

Beyond vocalizations, we also explore whether elephants produce other types of signals in conjunction with rumbles. We manually examine the presence of physical movements, such as locomotion, trampling, or stamping, within a window of ±50 s before and after each rumble. Such signals are visually identified when typical footfall signals (Figure 2A) are present within the time window and marked with *yes*. In case the rumble is isolated from motion‐typical signals, it is marked with *no*. In some instances, we could not reliably identify the signals and marked it with *not sure* (see Figure 3B).

### 2.2 CNN Input Data and Architecture

**FIGURE 4:**
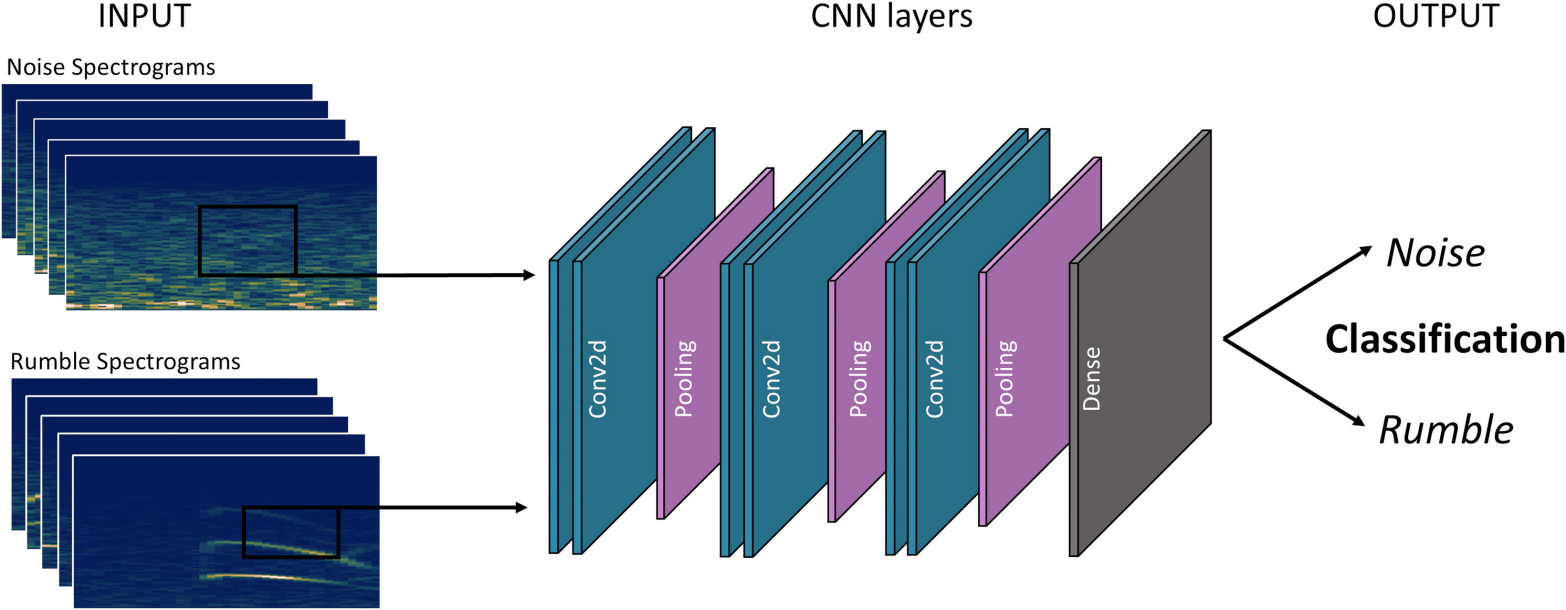
Schematic overview of the convolutional neural network (CNN) architecture, illustrating the input spectrograms, the layered structure for feature extraction and classification, and the final binary output distinguishing rumbles from background noise. Further examples of seismic and infrasound noise and rumble spectrograms are given in the supplements (Figure S4).

Manual picking of rumbles is time consuming and poses potential for human errors. Also, manual picking does not allow for near‐real time applications. Hence, we also test using convolutional neural networks (CNNs) to classify spectrograms of noise and rumbles (Figure 4) and evaluate their performance in cross‐domain applications. Noise is defined as all signal time segments that do not contain elephant rumbles, including environmental vibrations, anthropogenic activity, and sensor‐specific background signals. Since the objective of this study is robust rumble detection rather than the identification of individual noise sources, all non‐rumble signals are intentionally grouped into a single class. Noise samples were extracted across the full 30‐day recording period, covering both daytime and nighttime conditions. This ensures that the CNNs are exposed to the strong diurnal variability in background noise observed at the zoo and are trained to identify rumble‐specific spectral patterns rather than time‐dependent noise characteristics. Specifically, we train three separate CNNs, one on seismic spectrograms (S‐CNN), one on infrasound spectrograms (I‐CNN), and a third on combined data sets (C‐CNN) and apply each network to classify data from both domains. The spectrograms of noise and rumble snippets are represented as low‐resolution RGB images (232 × 115 pixels, 30 dpi), as we find optimized CNN performances at 30 dpi compared to higher or lower resolutions (Figure S5). The spectrograms are calculated from 12 s timeseries, with the picked timestamp of the rumble in the temporal centre. For the S‐CNN, 837 available rumble spectrograms are used, while for the I‐CNN, 1387 spectrograms are utilized. To find the best set of hyperparameters, we performed a grid search employing various constellations of filter size, dropout, batch size, and learning rate (Figure S6). The most sufficient set up consists of three convolutional blocks for feature extraction: the first consists of two Conv2D layers with 32 filters (of size 5 × 5), followed by MaxPooling2D (2 × 2) and 25% dropout. The second block contains two Conv2D layers with 64 filters (3 × 3), MaxPooling2D (2 × 2), and 25% dropout. The third block contains two Conv2D layers with 128 filters (5 × 5), MaxPooling2D (2 × 2), and 25% dropout. The extracted features are flattened and passed through a dense fully‐connected layer with 128 neurons and ReLU activation function, followed by 40% dropout. The convolutional neural network is trained using the RMSprop optimizer with a learning rate of 0.0005 and *ε* = 1e‐8. A batch size of 8 is used throughout all experiments. Early stopping is after 30 epochs to prevent overfitting and safe computational costs. The loss function is sparse categorical cross‐entropy. The final outputs are class probabilities. Hence, the output is a binary classification. 70% of the spectrograms are used for training, 10% for validation and 20% for tests, to ensure sufficient spectrograms not involved in training or validation. This configuration is used for all training to enable comparability between modalities.

## 3 Results

### 3.1 Comparison and Temporal Patterns of Infrasound and Seismic Signals

**FIGURE 5:**
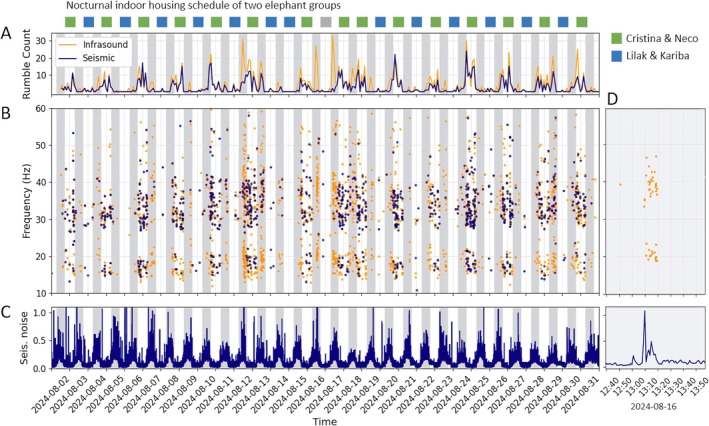
Elephant rumble activity in August 2024: (A) Rumble count for two‐hour windows of recordings of infrasound (yellow) and seismic detections (blue). (B) Temporal distribution and frequency of detected seismic and sound events. (C) Average seismic noise levels increase during the zoo's opening hours (indicated by gray bars). (D) Instances of major rumble activity on August 16 correlated with increased seismic amplitude, suggesting movement such as locomotion or trampling occurring concurrently with the rumble activity. The housing schedule on top of the figure shows overnight indoor housing (elephant house, see Figure 1) of Cristina and her male calf Neco in green and indoor housing of Lilak and Kariba (two adult cows) in blue. Consistent throughout the month, rumble activity significantly increases when Cristina and Neco are housed indoors overnight.

In Figure 2 we compare exemplary seismic and sound recordings. Footfall signals, generated by locomotion of elephants, are characterized by relatively sharp spikes in the seismogram and by a broad frequency range. These signals are exclusively observable in the seismic data (Figure 2A). In contrast, rumbles exhibit a tone‐like characteristic and are typically detectable in both infrasound and seismic data (Figure 2A,B). The spectrogram of sound data (Figure 2D) exhibits a lower noise level than the seismic sensor (Figure 2C), more sensitive to external noise sources. During zoo hours, from 7:00 am to 5:00 pm UTC (9:00 am to 7:00 pm local summertime), seismic noise significantly increases by a factor 5–10 due to the nearby restaurant, elephant facilities, nearby traffic, and visitors (Figure 5C). The seismic noise is often characterized by distinct monochromatic frequencies (e.g., 12 Hz, 25 Hz and 30 Hz), caused by nearby machines located close to the elephant facilities. Furthermore, shorter‐term broadband signals frequently occur during the day and background noise tends to be elevated at lower frequencies below 20 Hz. Noise recorded with the infrasound sensor exhibit rather broadband signals than transient signals (see Figure S4 in supplements). Generally, we noticed that the infrasound recordings are significantly less affected by increase in noise during the day (Figure 2E,F). The elevated daytime noise of seismic recordings results in fewer detectable seismic events than infrasound events during day. In particular, the visibility of the fundamental frequency of seismic rumbles is often missing due to increase noise at frequency < 20 Hz. However, nocturnal activity is detected sufficiently with both sensors. Sounds sensors measuring local changes of the air pressure are not sensitive to motion‐induced signals, highlighting the advantage of using seismic recordings along with infrasound sensors in such a noisy environment.

The detected rumbles have a characteristic tonal spectrum, typically manifesting a fundamental frequency between 10 Hz and 25 Hz, with a first harmonic observed between 25 Hz and 45 Hz (Figure 1D). Although the second harmonic occasionally appears at higher frequencies above 45 Hz, it is less prominent in the spectrograms, likely due to stronger attenuation of higher‐frequency waves. Depending on the detectability and source distance of the rumble and locomotion, the amplitude of the corresponding ground vibration velocity typically measures from 1 up to 10 micro‐meters per second. An exemplary day plot of seismic and infrasound data (Figure 2E,F) shows significant increase of seismic noise during the opening hours of the zoo, while infrasound exhibits a lower increase of noise during day time relative to acoustic background noise to night time.

The duration of individual rumbles varies, typically lasting between 1 and 8 s, with only a few exceeding the 8‐s mark (Figure 1B). On average, the duration is approximately 4 s. There is a subtle trend suggesting that rumbles within the range of the first harmonic tend to be longer when they occur at lower frequencies (Figure S7). The first harmonic, spanning from approximately 25 Hz–45 Hz, generally provides the most distinct and detectable signal in the data. However, there are instances where rumbles are observable solely at the fundamental frequency (10–25 Hz), underscoring the variability in spectral clarity across different harmonic levels. Comparing infrasound and seismic signals characteristics, we find that the duration as well as dominant frequency is consistent within the detections (Figure 1B). A corresponding analysis yielding > 0.97 (1 is perfect) of histogram similarity is provided in the Figure S8. This suggests a strong coupling between the generation of rumble calls as both sound and seismic waves, proving the ability of seismometers to capture the key features of such elephant vocalizations.

The result of picking rumbles by hand is a catalog containing over 2300 individual events, corresponding to 1387 elephant rumbles recorded across the month‐long period. 839 of the 1387 rumbles were detectable in both seismic data and infrasound data.

Over the 30‐day observation period, we observed significant temporal variability in the number of events (Figure 5A,B). The rumble counts exhibited a noticeable pattern, with systematically increased counts of 10–30 rumbles occurring approximately every second night (Figure 5A). This pattern can be explained by alternating indoor housing of different elephant sub‐groups, with one group showing significantly higher rumble activity. We discuss this in detail in the discussion section. Additionally, periods of sudden above‐average heightened rumble activity were primarily observed in infrasound data during opening hours (one example is depicted in Figure 5D). However, such peaks in activity were limited to specific dates—August 12, 16, and 17—suggesting particular events or interactions, e.g., with visitors that may have triggered these sequences of elephants' calls.

The observed periods of heightened activity were marked by clusters of multiple rumbles occurring within minutes, indicating a surge in elephant vocalizations (Figure 5D). In some cases, up to 20 rumbles appear in sequences with just a few seconds of intermediate time (see example in Figure S1). These phases of intense activity predominantly occur during the day, potentially triggered by interaction with visitors or between the mammals. Although detecting seismic events is challenging during these high‐activity periods due to general high seismic noise during the day, a noticeable short‐term increase in seismic signal amplitude of a factor of 10 or more suggests body motions, such as locomotion or trampling, often accompanying these rumbles (as shown exemplarily in Figure 2C). The observed correlation between heightened seismic signal amplitudes, mainly caused by the motion‐induced signals, and increased number of rumble events implies a combined vocal and physical sequence of activity during these episodes.

**FIGURE 6:**
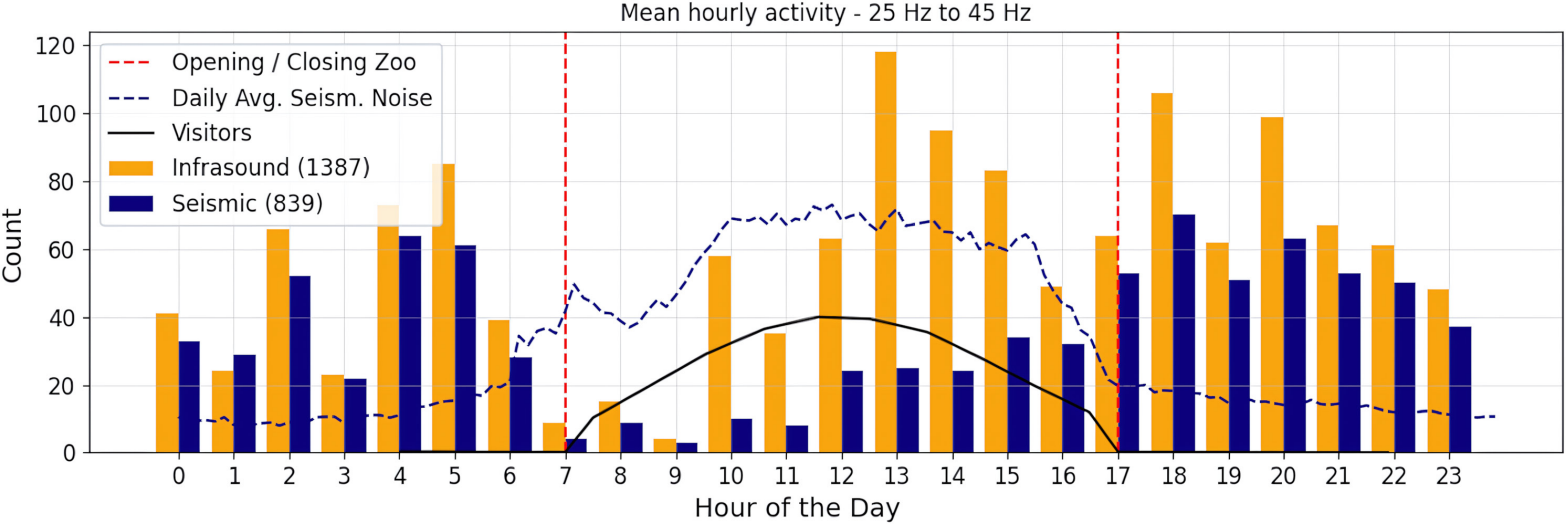
Hourly mean rumble activity averaged over 30 days. The mean seismic noise (blue dashed line) and estimated visitor numbers (black solid line) are shown alongside the zoo's opening and closing times (red dashed lines). Mean seismic noise values represent the average across all 30 days of recordings, while visitor numbers are derived from Google Maps statistics.

To further understand daily activity patterns, we categorize the rumbles by hour of occurrence each day, incorporating all data from the 30 observation days (Figure 6). We observe significant rumble activity throughout the night, with a noticeable distinct drop around 3:00 AM. As the zoo's opening time of 7:00 am approaches, the activity decreases significantly until 9:00 AM. Following this lull, activity begins to increase, peaking around 1:00 pm, likely corresponding with the increase in zoo visitors as the day progresses. As the zoo nears closing time at 5:00 pm, activity tends to decrease again. The comparison between seismic detections and infrasound detections along with the mean seismic noise plotted in Figure 5 shows that significantly more infrasound events are detected during opening hours, while a similar number of detections is observed during the night.

### 3.2 Rumble Sequences and Locomotion

Our findings reveal that the elephants frequently produce multiple rumbles in succession, with a temporal delay of 5 s or less in most cases. Events with a time difference of more than 5 s and less than 120 s occur less often. Nevertheless, isolated rumbles without a significant following rumble within 120 s are the majority. Overall, approximately half of the rumbles are followed by another rumble in less than 2 min, which likely indicates interaction between the five mammals across their enclosures (Figure 3A).

Additionally, we observe that in approximately two‐thirds of the rumbles, other forms of increased seismic signal amplitudes coincide with the vocalization, indicative of accompanying body motions. These might occur either before, during, or following the rumble. In a limited number of cases, ambiguity arises regarding the origin of the observed seismic signal, leaving open the possibility that some seismic signals might not stem directly from the elephant itself. However, such instances do not significantly detract from the overall patterns we identify (Figure 3B).

### 3.3 Automatic Rumble Detection Using CNN

To evaluate the automation of rumble and noise classification, we train three convolutional neural networks (CNNs). One using seismic rumbles and noise (S‐CNN), another using infrasound rumbles and noise (I‐CNN), and a third using combined data sets (C‐CNN).

**FIGURE 7:**
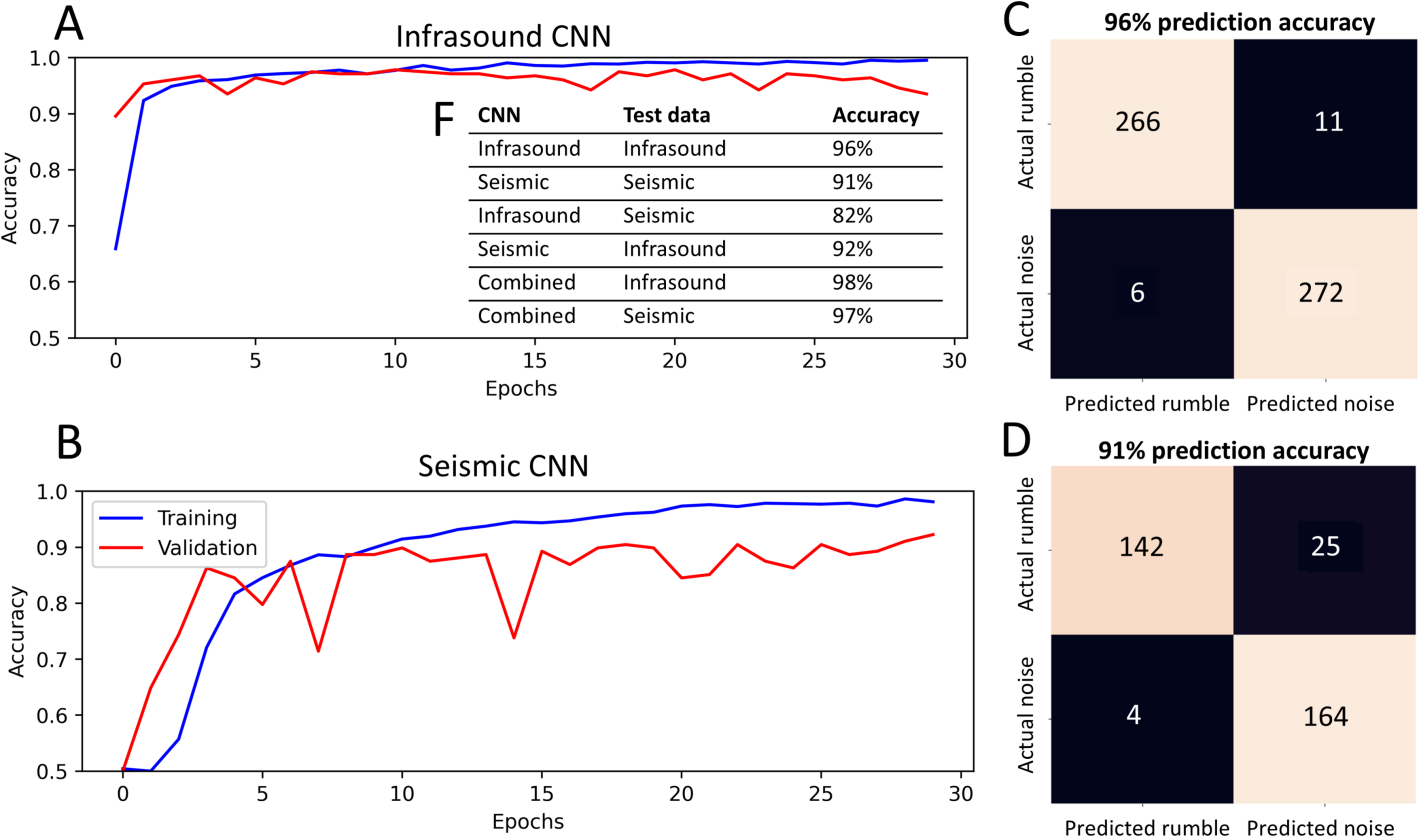
Training and validation accuracy for the I‐CNN (A) and S‐CNN (B) demonstrate stable accuracy convergence after 21 and 30 epochs, respectively. The confusion matrices for the test datasets show that the I‐CNN achieves a classification accuracy of 96% (C), while the S‐CNN reaches 91% accuracy (D). Accuracies for noise and rumble classification, including results from cross‐domain applications are given in the table (F).

We test both models on a test dataset comprising 20% of the total available rumbles, which the networks do not see during training. The I‐CNN achieves an accuracy of 96%, and the S‐CNN reaches 91%, demonstrating strong performance in distinguishing between noise and rumbles in their respective domains (Figure 7C,D).

Cross‐domain testing shows that the I‐CNN classifies seismic signals with 82% accuracy, while the S‐CNN classifies infrasound signals with 92% accuracy (Figure 7F). The C‐CNN achieved an accuracy of 98% on the infrasound test data and 97% on the seismic test data. Interestingly, despite being trained on fewer samples (837 for seismic compared to 1387 for infrasound), the S‐CNN performs more robustly on cross‐domain data. This robustness likely stems from the higher noise levels and variety in seismic recordings, often including transient signals from nearby vibrational sources or motion‐induced signals produced by the elephants. Also, the best result is obtained when combining seismic and infrasound for training (C‐CNN) and applied on infrasound‐only test data. In general, the contribution of seismic data helps to increase data and generalize more effectively when applied on infrasound data, while the I‐CNN fails to generalize sufficiently when applied to seismic signals.

## 4 Discussion

This study presents a catalog of more than 1350 rumbles recorded within 30 days using infrasound and seismic sensors in a zoo. While detailed behavioral interpretations are beyond the scope of this study, our focus is on comparing infrasound and seismic detectability as well as characteristic patterns of rumble activity.

### 4.1 Combining Infrasound and Seismic Data

The complementary seismic and infrasound datasets used in this study highlight the necessity of simultaneous recordings to capture a comprehensive view of elephant vibrational activity and behaviors (see Table 1). Nevertheless, the seismometer is located close to facilities and the restaurant. Other locations, e.g., on the other side of the enclosure, would likely be less affected by noise and provide increased signal‐to‐noise ratios. Our observations show that many rumbles are accompanied by motion‐induced signals, such as locomotion. The consistent association of these non‐vocal signals with rumbles indicates a versatile behavior, where elephants combine seismic and acoustic signals to convey messages and locomotion, possibly enhancing the efficacy of communication across varying distances and conditions. Nevertheless, all detected seismic rumbles were simultaneously recorded by the infrasound sensor, which is why we cannot confirm seismic‐only wave emission. Previous studies (O'Connell‐Rodwell et al. 2006; Mortimer et al. 2018) have already discussed the benefits of seismic waves in analyzing, localizing, and classifying signals from elephants and other wildlife (Reinwald et al. 2021; Steinmann et al. 2025), though experimental seismic datasets from zoos with specific conditions other than in natural environments remain scarce. Our study tackles the lack of such datasets by combining both infrasound and seismic recordings.

**TABLE 1:** Comparison of infrasound and seismic detection methods used for monitoring elephant activity, highlighting key characteristics, benefits, and limitations of each sensor in a zoo environment.

Feature	Infrasound characteristics	Seismic characteristics
Frequency Range	10–25 Hz (fundamental), harmonics above 25 Hz	
Duration	1–8 s, 4 s on average	
Signal propagation	Airborne acoustic pressure waves	Elastic waves (ground motion)
Motion‐induced signals	Not detectable	Clearly detectable
Rumble signals	Clearly detectable	Detectable, when not covered by noise
Noise (Daytime)	Low	High, due to noisy location
Noise (Night)	Low	Low
CNN accuracy	Min. 90%, insufficient for cross‐domain appl.	Min. 90%, also for cross‐domain appl.
Benefits	Low noise, clear rumble detections	Rumble & motion‐induced signal detection

Supported by the observation that numerous rumbles are followed by additional vocalizations (Figure 3), potentially from other elephants, it is highly likely that communication or interaction within the enclosure drives many of the observed rumbles. Previous studies (e.g., Pardo et al. 2023; O'Connell‐Rodwell et al. 2024) in natural environments have shown that such sequences can be associated with specific commands and reflect group hierarchies or social patterns (Poole et al. 1988, 2005). Furthermore, these sequences may be expressions of their emotional state (e.g., stress or joy), having the potential to provide information about their welfare when monitored, as demonstrated here.

Additionally, a particle motion analysis (Figure S2 in supplements) suggests that different frequency components of elephant rumbles may couple into the ground as distinct surface wave types. The first harmonic (30–40 Hz) predominantly shows horizontal polarization, consistent with Love wave propagation, while the fundamental frequency (16–20 Hz) exhibits strong vertical polarization indicative of Rayleigh waves. This finding reinforces the idea that elephants generate complex vibrational signals involving both vertical and horizontal ground motions, possibly enhancing communication effectiveness across variable terrain. At this stage of the research, it cannot be fully ruled out that ground‐coupled acoustic signals converted into seismic waves contribute to, or even dominate, the seismic recordings. Acoustic signals generated by the elephant may be transmitted into the subsurface and subsequently detected by the seismic sensor. Distinguishing between seismic signals directly induced by the elephant and those generated through acoustic coupling would require a dense sensor network deployed in close proximity to the animal, which was beyond the scope of this study. Future studies should leverage these characteristics to gain deeper insight into the physical coupling and propagation of seismic waves produced by elephant vocalizations.

### 4.2 Diurnal and Nocturnal Activity Patterns

Besides the connection observed between visitor presence and elephant vocalizations, routine training sessions and guided visitor interactions between 12:00 and 13:00 may further stimulate vocalizations. While this differs from natural habitats, it underscores the likely influence of the zoo environment on elephant activity. Notably, we could not establish a clear correlation between the total number of daily visitors during August and rumble counts (Figure S3). This suggests a potential saturation effect, which means although activity increases with rising visitor numbers during the day, it does not necessarily correlate with the absolute number of visitors. Hence, we exclude a relevant influence by overall visitor numbers on their activity. The observed activity deficit between 7:00 and 9:00 a.m. remains unresolved but is likely linked to feeding routines, the separation of elephants into smaller groups, such as separating the bull from the rest of the group, or is an effect due to the elephants leaving the indoor facility after the night and reducing their activity afterwards.

Considering that elephants typically sleep for approximately 2–4 h per day (Soltis et al. 2016; Gravett et al. 2017), and the Opel‐Zoo houses five elephants, it is unlikely to observe synchronized sleep periods among them. This also explains the continuous activity observed during night and most parts of the day. Moreover, as Mulder et al. (2024) suggest, distinguishing between nighttime and daytime activity is relevant, given that hot weather and global warming are likely to affect the diurnal and nocturnal behavior of elephants, with increased activity shifted to cool summer nights. Interestingly, we observe a significant increase in activity every second night, while activity noticeably diminishes on alternating nights (Figure 5A). Based on information provided by the elephant keepers, the observed pattern can be explained by the alternating indoor housing schedule of the elephants. Neco, the male child (4 years old), and his mother, Cristina, stay indoors approximately every second night. As a result, a significantly higher activity level in those nights is exhibited compared to the other night when the second group, which consists of two adult female elephants, is housed inside. The explanation for this behavior is not fully clear at the current state of research; however, according to information from the zoo keepers, one group is very likely communicating with the bull, separated from the others overnight.

Housing‐dependent activity patterns regarding walking and feeding are shown by Posta et al. (2013), confirming our findings; however, rumbles were not analyzed in their study. The clearly observed connection between wave‐based activity monitoring and individual elephant behavior highlights the need for further long‐term observations using more sensors to locate signal sources and to refine interpretations of the observed data. Moreover, the presented monitoring approach remains effective at night, unlike conventional cameras, which fail to detect rumbles or motions, specifically in the absence of infrared capability.

### 4.3 CNN‐Based Automated Rumble Detection

The general approach of using spectrograms for classification is inspired by bioacoustic applications (e.g., Schneider et al. 2024) and it proves to be highly efficient, offering low computational costs, a simple input format with minimal preprocessing, and high‐accuracy rumble classification. A collection of seismic and infrasound rumble and noise spectrograms is provided in Figure S4 in the supplements. The variety of noise in seismic recordings makes the S‐CNN more robust and improves its capability for generalization, although less input data was available. Costa et al. (2023) applied machine learning techniques with extensive prior spectrogram preprocessing to detect elephant rumbles, achieving accuracies of up to 97%. In contrast, our approach intentionally avoids such preprocessing and still attains comparable performance. Overall, the CNN performance could likely be enhanced by incorporating substantially larger datasets, such as public archives or additional recordings from natural habitats and other zoological institutions, or by further optimizing the neural network architecture. We also tested alternative methods including ratios of spectral bands similar to whale detections (inspired by Plourde and Nedimović 2022), spectrogram similarity analysis, and cross‐correlation between infrasound and seismic time series. Althoug all techniques could capture some of the rumbles correctly, the CNNs outperformed such conventional approaches. Training the models took less than 10 min on a single GPU of a standard notebook, making this approach widely accessible and practical without the need for high‐performance computing, advanced preprocessing, or extensive training time. The strong performance of the relatively simple CNN used in this study is consistent with the findings of Szenicer et al. (2021), who evaluated both one‐dimensional and two‐dimensional CNNs developed from scratch as well as pretrained models. They demonstrated that pretrained networks are particularly well suited for environmental sound classification, suggesting that such models could further enhance the robustness of our CNN‐based approach. In contrast, Steinmann et al. (2025) employed a hybrid method combining a scattering transform based on seismic signal wavelets with a support vector classifier for feature extraction and classification of elephant footfalls. In future work, a similar approach could facilitate the automatic detection of elephant footfalls in zoo environments; however, its implementation lies beyond the scope of the present study.

The developments presented in our study enable the application of the trained neural network to continuous recordings by segmenting time series into spectrograms and classifying their content. Future research should explore unsupervised clustering techniques on these spectrograms to identify similarities and differences among rumbles, which could progress decoding their meaning or attributing them to individual elephants. However, these considerations are beyond the scope of the present study.

## 5 Conclusion

In this study, over 1350 rumbles from African elephants in a zoo were detected and analyzed over a 30‐day period using co‐located infrasound and seismic sensors. We find that rumbles are generally clearer in sound data than seismic recordings due to lower noise levels specifically when the zoo is open. However, seismic recordings contain rumbles and motion‐induced signals such as locomotion, highlighting the benefit of combining both seismic and infrasound recordings to monitor the full range of wave‐based signals produced by elephants. A significant increase of rumble activity every second night can be assigned to the housing schedule of the elephant groups, which is a key influence on the observed rumble activity. This indicates animal‐specific activity patterns depending on the housing schedule. Moreover, observations indicate that rumbles are rarely isolated events; instead, they often occur in sequences and are mostly accompanied by body movements such as locomotion and trampling, as shown by seismic signals absent in sound recordings. As a proof of concept for automated monitoring, we trained and validated CNNs on spectrograms of rumbles and noise, achieving classification accuracies of up to 98%. Notably, CNNs trained with seismic data demonstrate greater robustness and generalization for cross‐domain applications.

The presented approach and results have the potential to advance the monitoring and understanding of elephant communication, behavior, and interaction in a controlled zoo environment, characterized by specific conditions and anthropogenic influences that are not present in natural habitats. Future studies should expand upon these findings by incorporating additional sensors and data sources (such as video footage), e.g., to enable precise localization, tracking and classification of elephant communication and locomotion within their enclosure. This would allow for further confirmation and refinement of the observations made in this study and could help to better monitor and understand the activity and welfare of elephants kept in zoos.

## Author Contributions

**Fabian Limberger:** conceptualization (lead), data curation (lead), formal analysis (lead), investigation (lead), methodology (lead), project administration (lead), software (lead), visualization (lead), writing – original draft (lead), writing – review and editing (equal). **Georg Rümpker:** conceptualization (lead), funding acquisition (lead), investigation (supporting), methodology (supporting), supervision (supporting), writing – review and editing (supporting). **Ronja Wesemann:** methodology (supporting), software (supporting), visualization (supporting). **Abolfazl Komeazi:** methodology (supporting), software (supporting), writing – review and editing (supporting). **Tanja Spengler:** project administration (supporting), writing – review and editing (supporting). **Martin Becker:** project administration (supporting), writing – review and editing (supporting).

## Ethics Statement

This research was conducted as a fully non‐invasive study, which did not require ethical reviews and approvals. All measurements were carried out in close coordination with zookeepers and other responsible personnel, adhering to the ethical standards and guidelines of the facility. The passive data collection method ensured no interference with management routines and did not disrupt the animals' usual behavior. The maximum frequency considered in this study was 80 Hz, thereby preventing the recording of human voices.

## Conflicts of Interest

The authors declare no conflicts of interest.

## Supporting information


**Figure S1:** ece373220‐sup‐0001‐Figures.zip.

## Data Availability

Spectrograms and code are publicly available via Github (https://github.com/flimberg/Limberger_etal_2026_SeismicSoundSignalsZooElephant) or can be requested from the corresponding author.
