# Supplementary material for: Monitoring Zoo Elephant Rumble Activity Using Combined Seismic and Acoustic Data
Source: Ecol Evol. 2026 Mar 8;16(3):e73220. doi: 10.1002/ece3.73220 (PMC12968057; doi:10.1002/ece3.73220)
Supplement: Supplementary file 1 — Figure S1: ece373220‐sup‐0001‐Figures.zip. [file ECE3-16-e73220-s001.zip › Elephant_rumbles_Limberger_etal_2025_supplements_revised1.docx]

Supplementary material to publication

Monitoring zoo elephant rumble activity using combined seismic and acoustic data

**
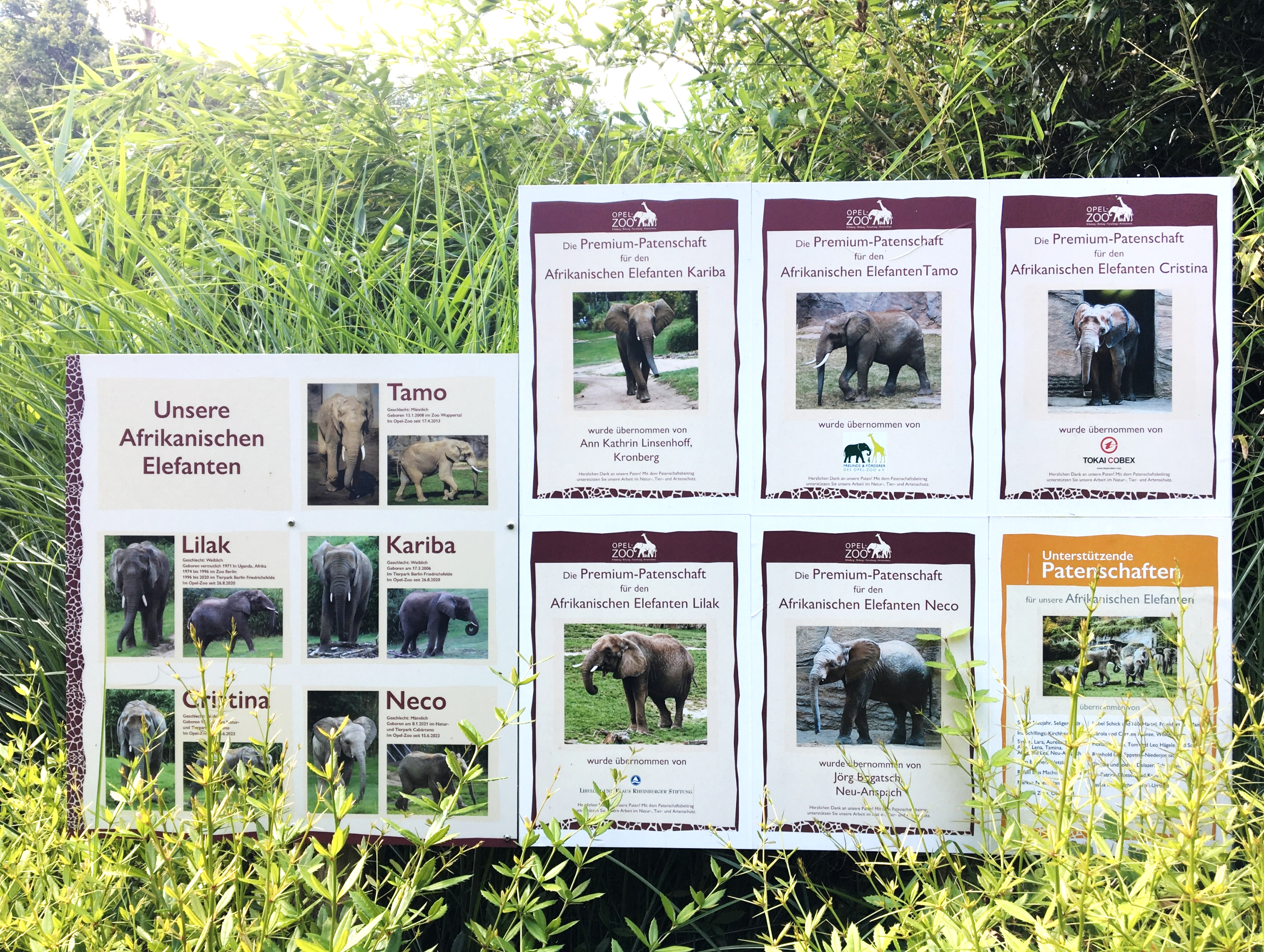
**

*Information about the elephants in the Opel-Zoo. (Picture taken August 2024).*

**
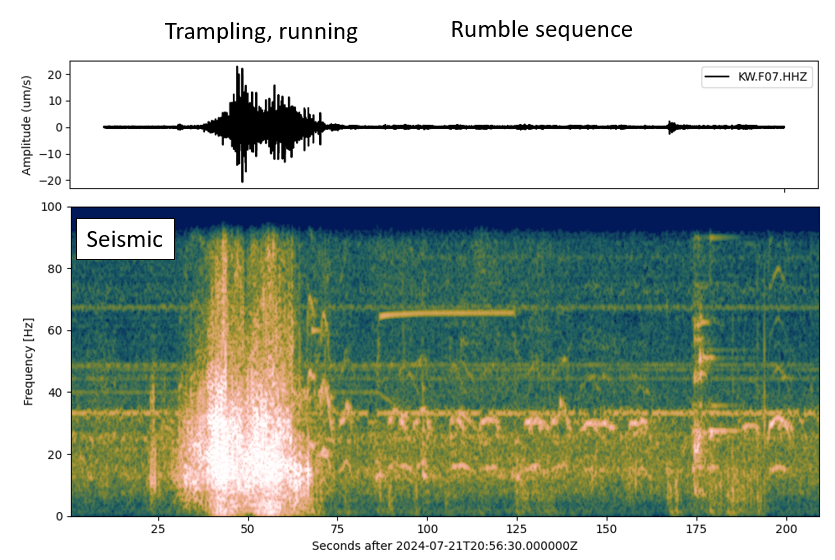
**

Figure S1: Example of a sequence of many rumbles following a strong motion-induced signal (e.g., locomotion, trampling).


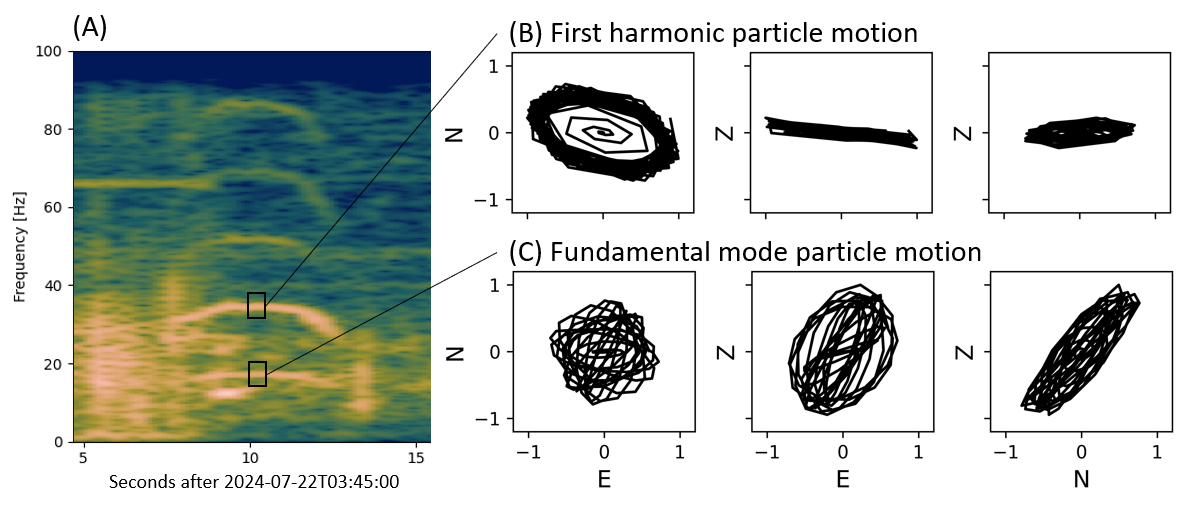


Figure S2: (A) Seismic Spectrogram of an exemplary rumble. (B) The particle motion of the first harmonic oscillation and (C) the fundamental frequency. Amplitudes are normalized to their respective maxima. Seismograms of one second duration are filtered to 16-20 Hz for the fundamental frequency and 30-40 Hz for the first harmonic, ensuring the capture of the relevant spectral range.


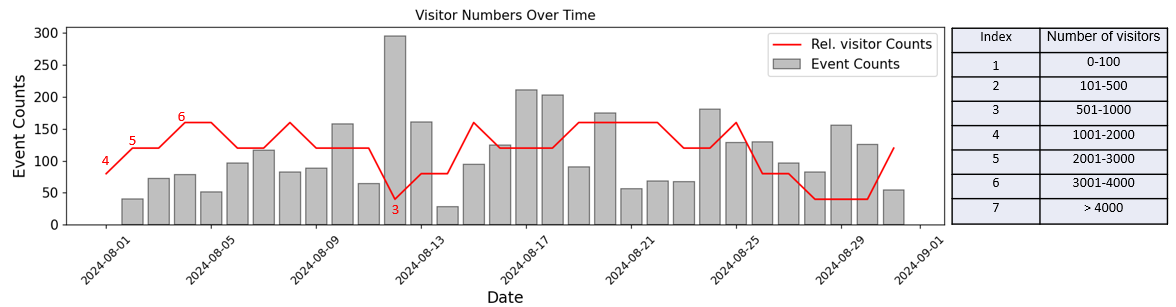


Figure S3: Comparison between event counts and visitor numbers. The numbers were provided as indices covering a specific number range. Explicit numbers were not provided due to data security. Days with high or low elephant activity does not correlate with high or low visitor number.

**
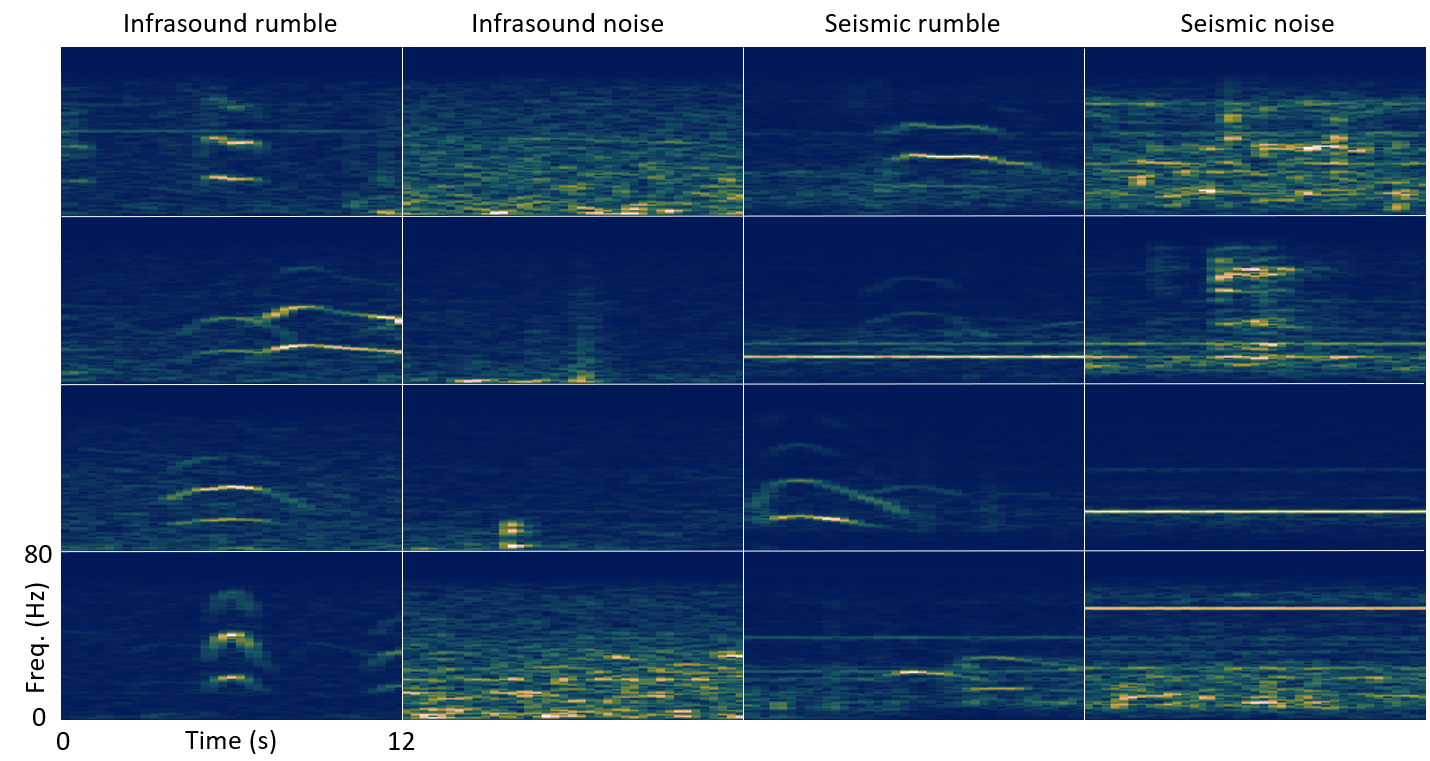
**

Figure S4: Examples of seismic and infrasound rumble and noise spectrograms serving as input for the CNN.

**
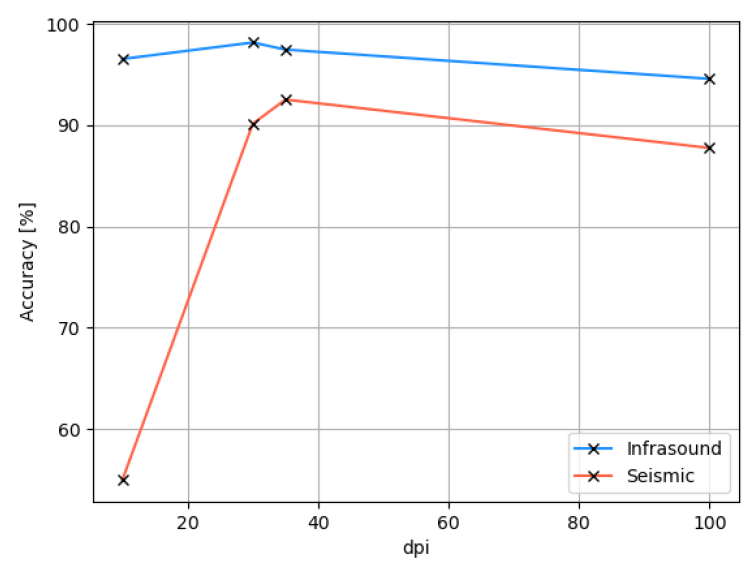
**

Figure S5: Prediction accuracy as function of spectrogram image resolution for seismic and infrasound data sets. Maximum accuracy is obtained at approximately 30 dpi.


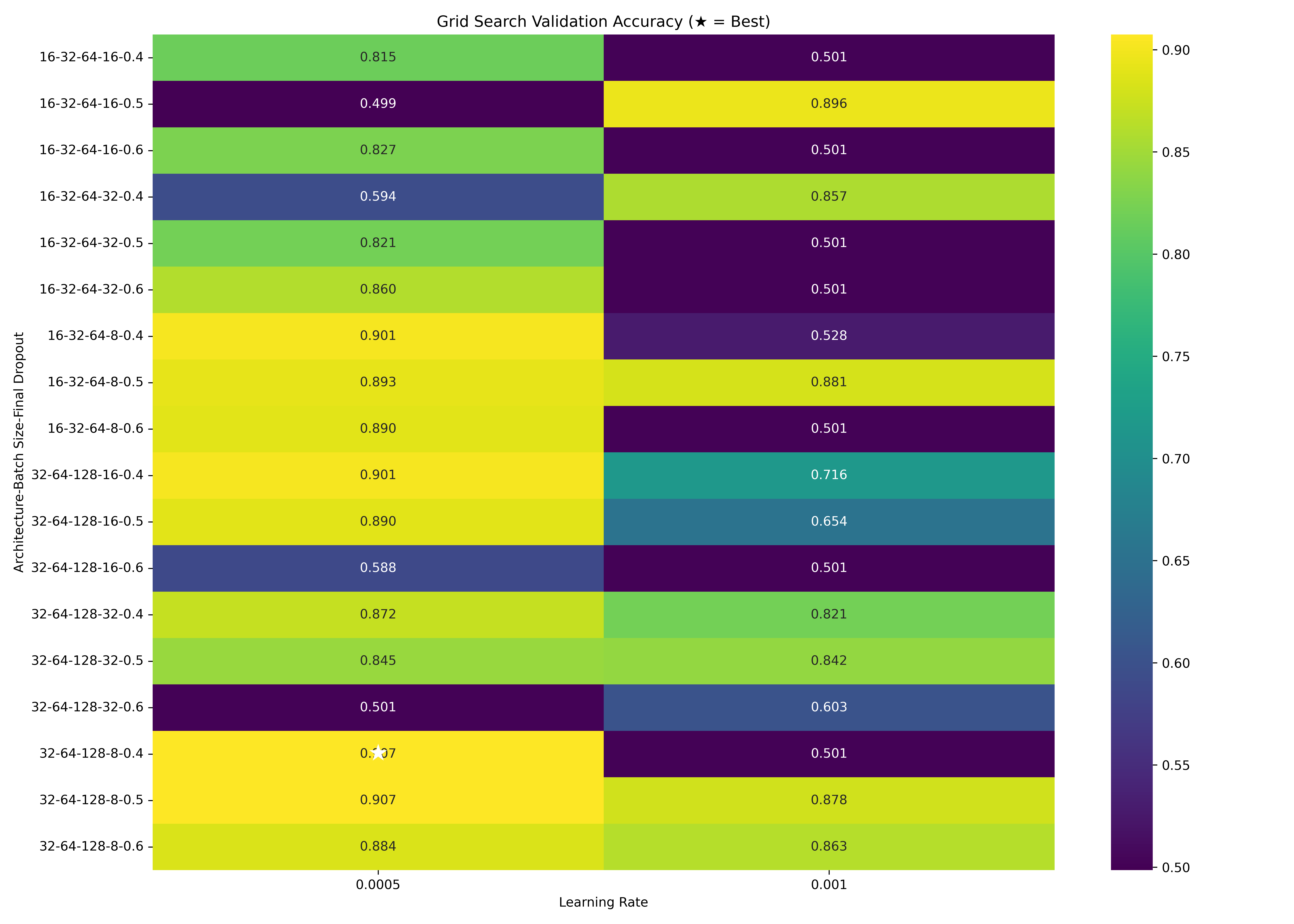


Figure S6: Results of accuracy grid search to find the most sufficient set of hyperparameters, based on seismic data and 10 epochs. We tested CNN-architecture design of 16-32-64 and 32-64-128, batch sizes of 8,16, and 32, dropout of 0.4, 0.5, and 0.6 and learning rate of 0.0005 and 0.001. Best results is obtained for 32-64-128, batch size 8, dropout 0.4 and learning rate 0.0005, which is used for all trainings in the study.


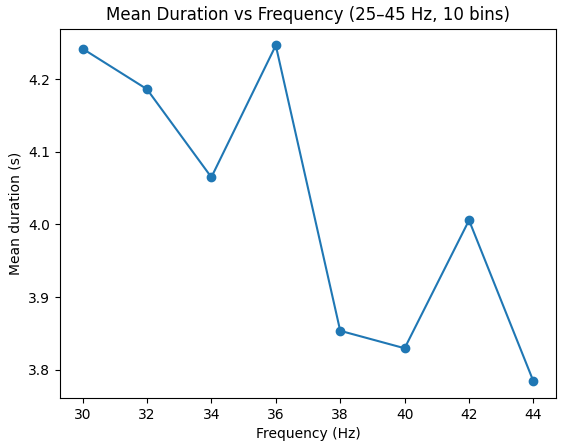


Figure S7: Mean rumble duration vs. frequency, based on infrasound data between 25 and 45 Hz (first harmonic) and binned into ten frequency groups.


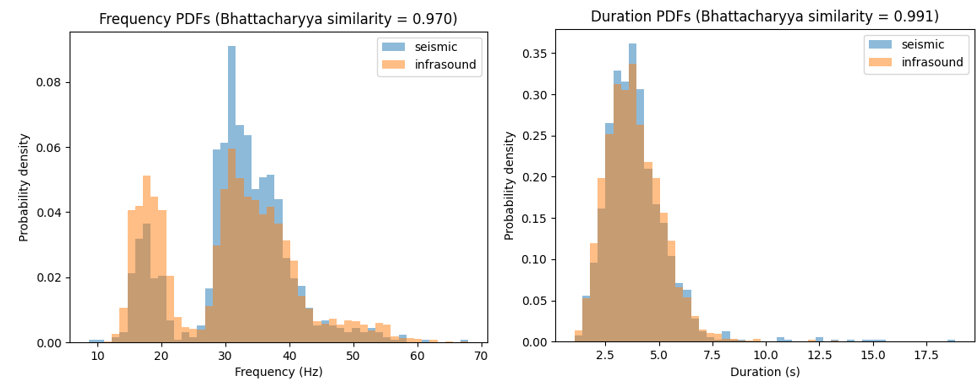


Figure S8: Quantification of similarity between seismic and infrasound density distribution regarding frequency and duration of the rumbles. Similarities are 0.97 (frequency) and 0.991 (duration), demonstrating strong similarity coefficient (=1 would be perfect) and hence correlation between seismic and infrasound data sets.

Reference:
Bhattacharyya, A. (March 1943). "On a measure of divergence between two statistical populations defined by their probability distributions". *Bulletin of the Calcutta Mathematical Society*. **35**: 99–109. MR 0010358.
