# Supplementary figures and images for: Monitoring Zoo Elephant Rumble Activity Using Combined Seismic and Acoustic Data
Source: Ecol Evol. 2026 Mar 8;16(3):e73220. doi: 10.1002/ece3.73220 (PMC12968057; doi:10.1002/ece3.73220)

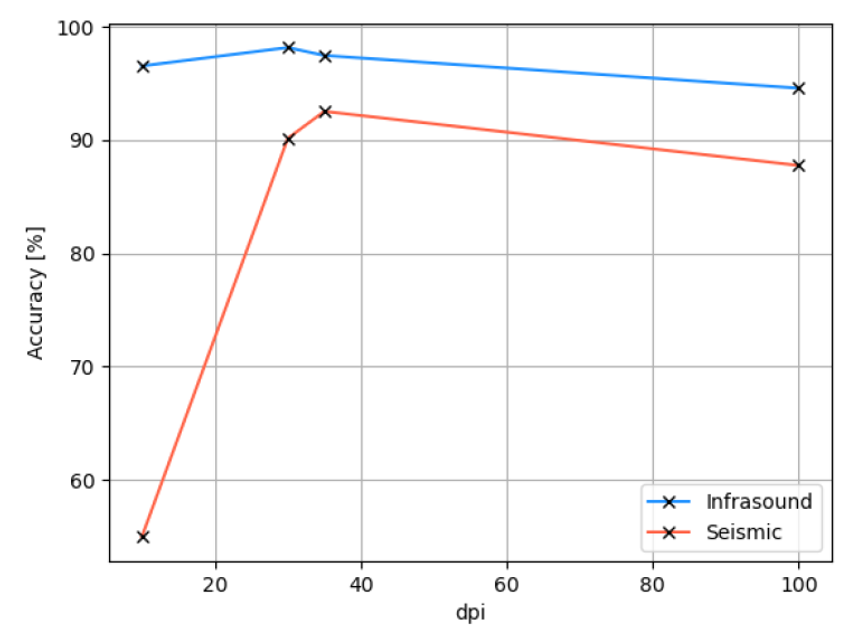

Supplement: Supplementary file 1 — Figure S1: ece373220‐sup‐0001‐Figures.zip. [file ECE3-16-e73220-s001.zip › ece373220-sup-0005-FigureS5@FigS5.png]

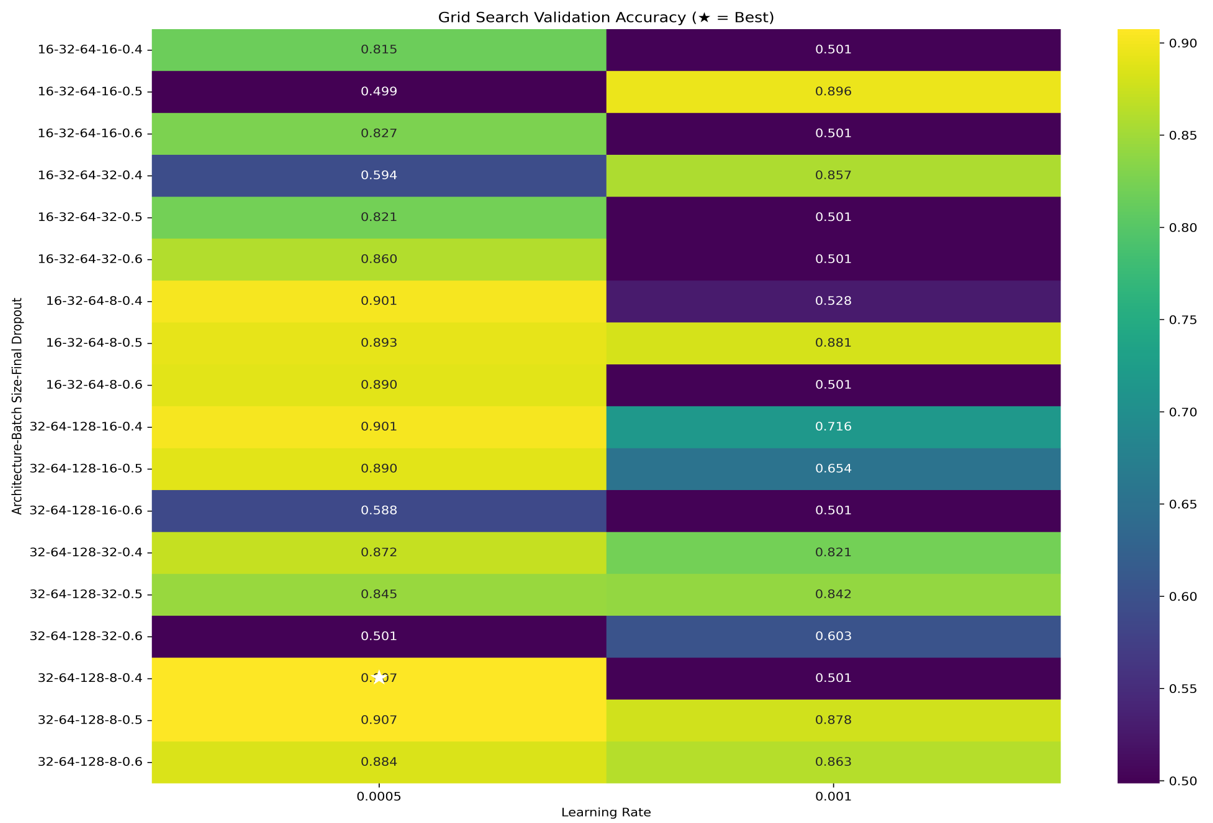

Supplement: Supplementary file 1 — Figure S1: ece373220‐sup‐0001‐Figures.zip. [file ECE3-16-e73220-s001.zip › ece373220-sup-0006-FigureS6@FigS6.png]

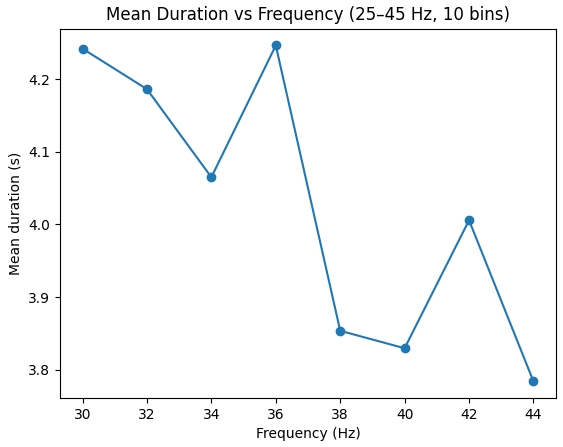

Supplement: Supplementary file 1 — Figure S1: ece373220‐sup‐0001‐Figures.zip. [file ECE3-16-e73220-s001.zip › ece373220-sup-0007-FigureS7@FigS7.png]

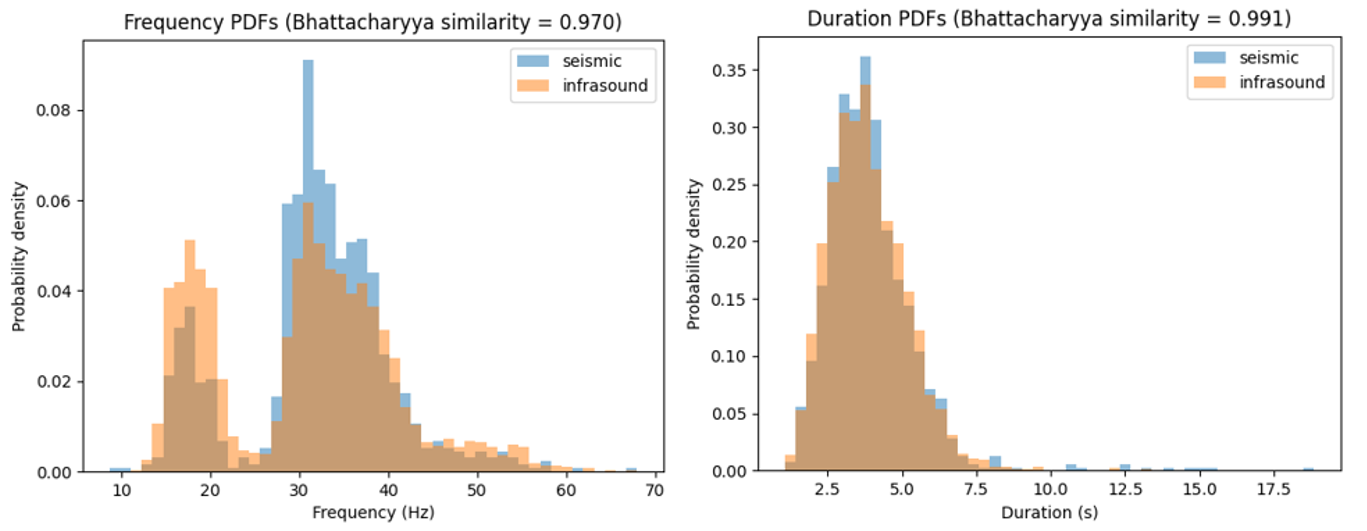

Supplement: Supplementary file 1 — Figure S1: ece373220‐sup‐0001‐Figures.zip. [file ECE3-16-e73220-s001.zip › ece373220-sup-0008-FigureS8@FigS8.png]

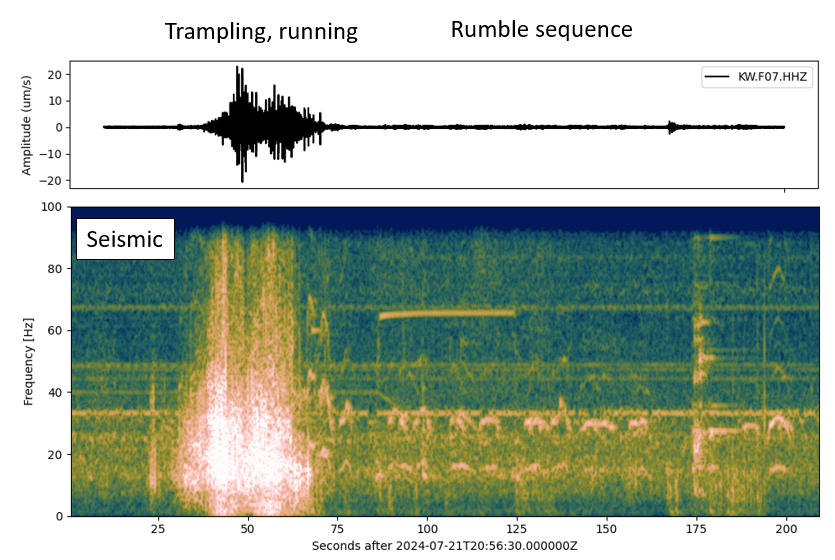

Supplement: Supplementary file 1 — Figure S1: ece373220‐sup‐0001‐Figures.zip. [file ECE3-16-e73220-s001.zip › ece373220-sup-0001-FigureS1@FigS1.png]

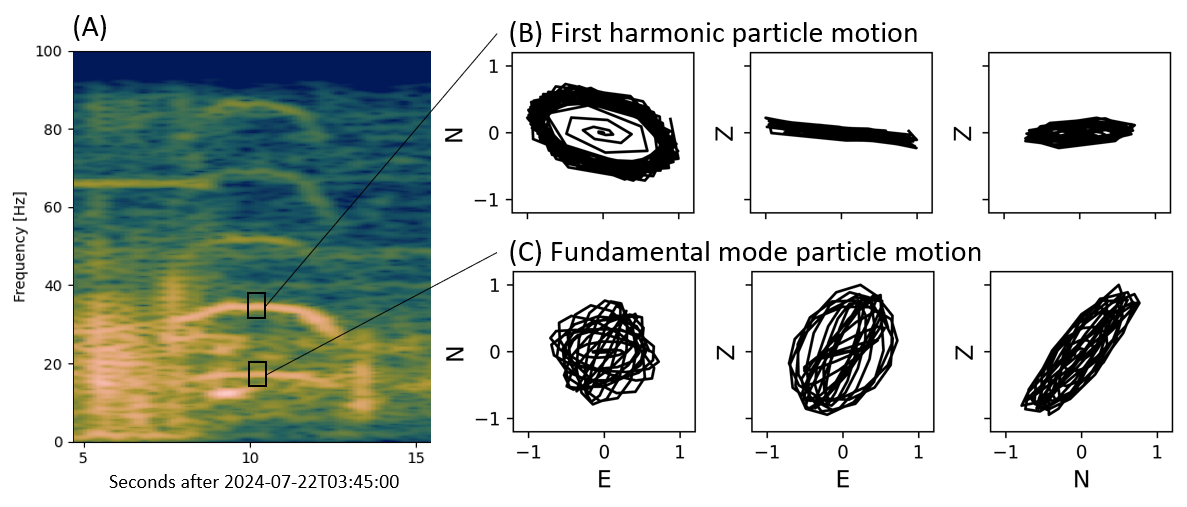

Supplement: Supplementary file 1 — Figure S1: ece373220‐sup‐0001‐Figures.zip. [file ECE3-16-e73220-s001.zip › ece373220-sup-0002-FigureS2@FigS2.png]

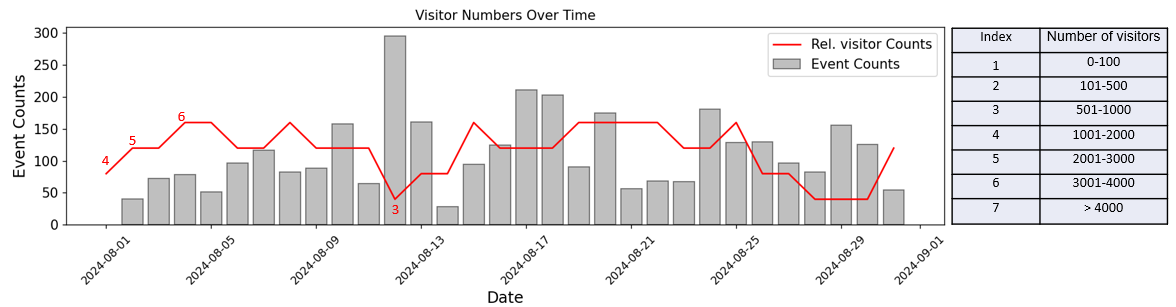

Supplement: Supplementary file 1 — Figure S1: ece373220‐sup‐0001‐Figures.zip. [file ECE3-16-e73220-s001.zip › ece373220-sup-0003-FigureS3@FigS3.png]

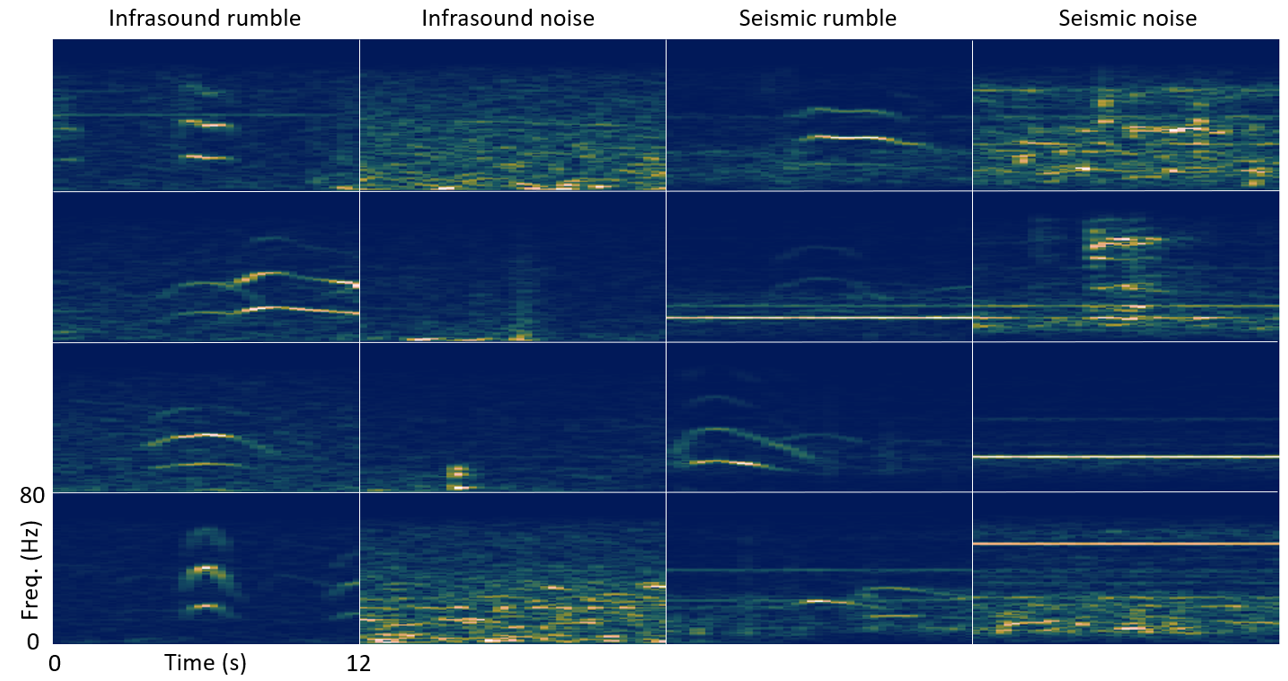

Supplement: Supplementary file 1 — Figure S1: ece373220‐sup‐0001‐Figures.zip. [file ECE3-16-e73220-s001.zip › ece373220-sup-0004-FigureS4@FigS4.png]
